# DIpartite: A tool for detecting bipartite motifs by considering base interdependencies

**DOI:** 10.1371/journal.pone.0220207

**Published:** 2019-08-30

**Authors:** Mohammad Vahed, Jun-ichi Ishihara, Hiroki Takahashi

**Affiliations:** 1 Medical Mycology Research Center, Chiba University, Chiba, Japan; 2 Molecular Chirality Research Center, Chiba University, Chiba, Japan; University of North Carolina at Charlotte, UNITED STATES

## Abstract

It is extremely important to identify transcription factor binding sites (TFBSs). Some TFBSs are proposed to be bipartite motifs known as two-block motifs separated by gap sequences with variable lengths. While position weight matrix (PWM) is commonly used for the representation and prediction of TFBSs, dinucleotide weight matrix (DWM) enables expression of the interdependencies of neighboring bases. By incorporating DWM into the detection of bipartite motifs, we have developed a novel tool for *ab initio* motif detection, DIpartite (bi**partite** motif detection tool based on **di**nucleotide weight matrix) using a Gibbs sampling strategy and the minimization of Shannon’s entropy. DIpartite predicts the bipartite motifs by considering the interdependencies of neighboring positions, that is, DWM. We compared DIpartite with other available alternatives by using test datasets, namely, of CRP in *E*. *coli*, sigma factors in *B*. *subtilis*, and promoter sequences in humans. We have developed DIpartite for the detection of TFBSs, particularly bipartite motifs. DIpartite enables *ab initio* prediction of conserved motifs based on not only PWM, but also DWM. We evaluated the performance of DIpartite by comparing it with freely available tools, such as MEME, BioProspector, BiPad, and AMD. Taken the obtained findings together, DIpartite performs equivalently to or better than these other tools, especially for detecting bipartite motifs with variable gaps. DIpartite requires users to specify the motif lengths, gap length, and PWM or DWM. DIpartite is available for use at https://github.com/Mohammad-Vahed/DIpartite.

## Introduction

Gene expression is often regulated by transcription factors (TFs). TFs bind to specific DNA-binding sites and modulate the expression of genes. Therefore, to understand transcriptional regulations, given its complexity, it is extremely important to make accurate inferences about transcription factor binding sites (TFBSs). High-throughput ChIP-seq, which is widely used to study TF–DNA interactions, provides the sequences of binding regions [1,2]. TFBSs can be determined as the most over-represented motif in a given set of DNA sequences.

Bipartite motifs are defined as extensions of one-block TFBSs, that is, two conserved motifs separated by variable gaps. Several different types of bipartite motifs have been proposed in both prokaryotes and eukaryotes [3,4]. Shultzaberger et al. (2001) proposed the bipartite model of ribosome binding sites, in which they are composed of a Shine–Dalgarno sequence and an initiation region in *Escherichia coli* [3]. In *Bacillus subtilis*, the principal sigma factor in vegetative growth, SigA, binds to the bipartite motif separated by variable gaps, TGACA<spacer>TATAAT [5–7]. Baichoo and Helmann (2002) determined the bipartite motif, TGATAAT<spacer>ATTATCA, of the ferric uptake repressor Fur [8,9]. It has been reported that the global regulator AbrB can recognize bipartite motifs [10–12]. As in the case of eukaryotes, the existence of bipartite motifs of yeast TFs, such as ABF1 and GAL4, has been confirmed [13,14]. It has been reported that around 30% of the promoter sequences contain bipartite motifs with constant gaps in humans [15]. The level of conservation of the motif M4 (ACTAYRNNNCCCR) was reported to be much higher than those for most known motifs. Similarly, the TFs CAR and RXR bind to bipartite motifs in humans [4]. Thus, it is conceivable that TFs work in a cooperative manner and recognize bipartite motifs to regulate gene expression [16,17]. Several tools such as BioProspector [18], BiPad [19,20], and AMD [21] are available for the *ab initio* prediction of bipartite motifs for a set of DNA sequences, while many tools have been developed for the prediction of one-block TFBSs, such as Consensus [22], Gibbs Sampler [23], and MEME [24]. BioProspector based on Gibbs sampling [18] and BiPad based on the entropy minimization method [19,20] enable the identification of bipartite motifs with variable gaps. AMD identifies bipartite motifs with constant gaps by comparing the target sequences with the background sequences regardless of whether the motifs are long or short, gapped or contiguous [21].

Position weight matrices (PWMs) are commonly used to find and represent TFBSs [25]. They are based on the assumption that each nucleotide independently participates in the TF–DNA interaction. However, it has long been known that interactions between neighboring DNA bases affect TF–DNA interactions. For example, a single amino acid interacts with multiple bases simultaneously [26]. Zhao et al. (2012) clearly showed the existence of dinucleotide dependency in TFs [27,28]. Indeed, PWMs perform well in modeling TFBS properties, but are inadequate for considering position interdependencies. There are interdependencies between neighboring positions of the binding sites of CRP and LexA in *E*. *coli* [29]. It has been reported that the method based on dinucleotide weight matrix (DWM) outperformed that based on PWM for yeast datasets [30]. In fact, Weirauch et al. (2013) observed an improvement of performance of motif detection upon incorporating dinucleotide interactions [28]. Although BioProspector and BiPad predict bipartite motifs, they are based on the assumption of independencies among bases, namely, PWM.

Here, we present a novel bipartite motif detection tool, DIpartite (bi**partite** motif detection tool based on **di**nucleotide weight matrix). DIpartite predicts the bipartite motif by considering interdependencies of neighboring positions, namely, DWM. We compared DIpartite with other available alternatives by using test datasets from prokaryote and eukaryote, namely, of CRP in *E*. *coli*, sigma factors in *B*. *subtilis*, and promoter motifs in humans.

## Materials and methods

### A novel method for predicting bipartite motifs by incorporating base-pair dependencies

DIpartite identifies the bipartite motifs with variable gaps based on PWM or DWM from the input sequences (S1 Fig). Since it is reported that the bipartite motif represents well by Shannon’s entropy [3,19,20], we set the objective function to minimize the entropy. Similar to BiPad [19,20], the algorithm of DIpartite is based on Gibbs sampling and the minimization of information content (IC) by a greedy algorithm. DIpartite adopts the Gibbs sampling strategy which initializes the motif positions for all input sequences at random, and iteratively improves the entropy of PWM or DWM by updating the motif position.

### Objective function

Input data have *N* sequences for prediction of the bipartite motifs separated by gaps. Similar to BiPad [19,20], the bipartite motifs are expressed as *l*_*L*_<*d*>*l*_*R*_, where *l*_*L*_ and *l*_*R*_ are the widths of left and right motifs, respectively, and *d* is gap length. We set the objective function to minimize Shannon’s entropy for PWM or DWM of the concatenated motif of the left and right motifs, in Eq 1:

$${\hat{M}}_{LR}={\mathrm{argmin}}_{M_{LR}}({IC}_{M_{LR}})$$ (1)

where *M*_*LR*_ is the concatenated motif, and ${IC}_{M_{LR}}$ is the entropy for the motif *M*_*LR*_. Here, ${IC}_{M_{LR}}$ is given by:

$${IC}_{M_{LR}}=\sum_{i}^{j}\sum_{x\in X}-p_{i}(x)\times \log\{\frac{p_{i}(x)}{b(x)}\},i=\left\{ \begin{array}{c} 1,PWM\\ 2,DWM \end{array},\right.X=\left\{ \begin{array}{c} \{A,C,G,T\},\;PWM\\ \{\mathrm{AA},\mathrm{AC},\cdots ,\mathrm{TT}\},\;DWM \end{array}\right.$$ (2)

where *p*_*i*_(*x*) and *b*(*x*) are the composition of *x* in the motif sites and the background sites (not motif sites), respectively. *x* is one of the mononucleotides or dinucleotides for PWM or DWM, respectively. *j* is the sum of the lengths of the left and right motifs. *p*_*i*_(*x*) and *b*(*x*) are given by:

$$p_{i}(x)=\frac{f_{i}(x)+\beta /k}{N+\beta },k=\left\{ \begin{array}{l} \;4,\;PWM\\ 16,\;DWM \end{array}\right.$$ (3)

$$b(x)=\frac{g(x)+\beta /k}{n+\beta }$$ (4)

where *N* is the total number of input sequences. *f*_*i*_(*x*) is the frequency of *x* at the position *i*, that is, the mononucleotide at position *i* for PWM, or the dinucleotide at position *i* −1 and *i* for DWM. *k* is the number of the patterns, that is, *k* = 4 for PWM or *k* = 16 for DWM. *n* is the total number of the mononucleotides for PWM or dinucleotides for DWM that are not located at the motif sites. *β* is the total pseudo-count. *g*(*x*) is the frequency of *x* in the background sites. We set *β* = 1.

### Overview of the algorithm

The algorithm of DIpartite works through an iterative process of calculating entropy. DIpartite is implemented in C++ and available under the CNU v3 license. Fasta and text formats are allowed as input files. Users can specify the lengths of the left and right motifs, the gap length, and PWM for the mononucleotide or DWM for the dinucleotide. The software works for OOPS (one occurrence per sequence), ZOOPS (Zero or one bipartite occurrence per sequence), or ANR (any number of repetitions).

### Performance evaluation

The nucleotide-level correlation coefficient (*nCC*) was used to evaluate the performance of each tools for the same input data [31]. *nCC* is given by:

$$nCC=\frac{nTP\times nTN-nFN\times nFP}{\sqrt{\left(nTP+nFN)(nTN+nFP)(nTP+nFP)(nTN+nFN\right)}}$$ (5)

where *nTP* is the number of nucleotide positions in both known sites and predicted sites, *nFN* is the number of nucleotide positions in known sites but not in predicted sites, *nFP* is the number of nucleotide positions not in known sites but in predicted sites, and *nTN* is the number of nucleotide positions in neither known sites nor predicted sites. We adopted the combined *nCC* by adding *nTP*, *nFN*, *nFP*, and *nTN* over the data sets.

### CRP

CRP binding sites in *E*. *coli* were retrieved from Regulon DB as “TF binding sites” (Release: 9.4 Date: 05-08-2017) [32]. For example, the motif sequences of two ECK125158203 entries were identical although the transcription unit was different, i.e., fumA and fumAC. Out of 374 sequences of CRP binding sites, 323 unique sequences ranging from 36 bp to 42bp were filtered and used for the performance comparison. The binding site lengths consisted of 16 bp (11 binding sites), 17 bp (one binding site), 20 bp (one binding site), 22 bp (308 binding sites), and 23 bp (two binding sites).

### Promoter motifs in human

Xie et al. [15] proposed the 1,460 motifs in human. We sought the motifs with the gap lengths greater than or equal to the lengths of left and right motifs. Among of them, we selected 46 motifs with more than 4-nt gaps as the test datasets of two-block motifs. The promoter sequences around the positions of each motifs (500 bp upstream to 500 bp downstream) were retrieved as the target sets.

### Sigma factor

As the dataset of bipartite motifs with variable gap lengths, the sigma factor dataset in *B*. *subtilis* from DBTBS [7] was used. The nine of the bipartite sigma transcription factors in *B*. *subtilis* were used. The minimum and maximum gap lengths of sigma factors were determined based on all identified binding sites: σ^A^ (344 sequences ranging from 38 bp to 93 bp, 6<[11,23]>6), σ^B^ (64 sequences ranging from 39 bp to 64 bp, 6<[12,18]>6), σ^D^ (30 sequences ranging from 44 bp to 57 bp, 4<[12,18]>8), σ^E^ (70 sequences ranging from 41 bp to 58 bp, 7<[12,18]>8), σ^F^ (25 sequences ranging from 41 bp to 71 bp, 5<[13,19]>10), σ^G^ (55 sequences ranging from 40 bp to 76 bp, 5<[15,20]>7), σ^H^ (25 sequences ranging from 41 bp to 60 bp, 7<[9,18]>5), σ^K^ (53 sequences ranging from 38 bp to 85 bp, 4<[9,17]>9), and σ^W^ (34 sequences ranging from 38 bp to 53 bp, 10<[13,17]>6).

### Other programs used for comparison

Four popular tools, namely MEME (ver. 5.0.3), BioProspector (release 2), AMD, and BiPad (ver. 2), were compared with DIpartite.

For the CRP dataset, MEME was executed with the options “-mod oops”, “-dna”, “-w 22”, “-minw 22”, and “-maxw 22”. BioProspector was executed with the options “-n 50”, and “-n 3”. AMD was executed with the options “-MI” and “-T 1”. BiPad was executed with the options “-l 22”, “-r 0”, “-a 0”, “-b 0”, “-i”, and “-y 1000”. AMD was executed with the option “-T 2” for two sigma datasets, i.e., σ^E^ and σ^F^. We used the background sequences for AMD: the 200 bp upstream regions of 4,314 genes in *E*. *coli* K-12 (NC_000913.3), the promoter sequences of all human genes (hg17: upstream1000.fa.gz), and the 200 bp upstream regions of 4,448 genes in *B*. *subtilis* 168 (NC_000964.3).

## Results

### Interdependencies of neighboring DNA bases in CRP

**Fig 1:**
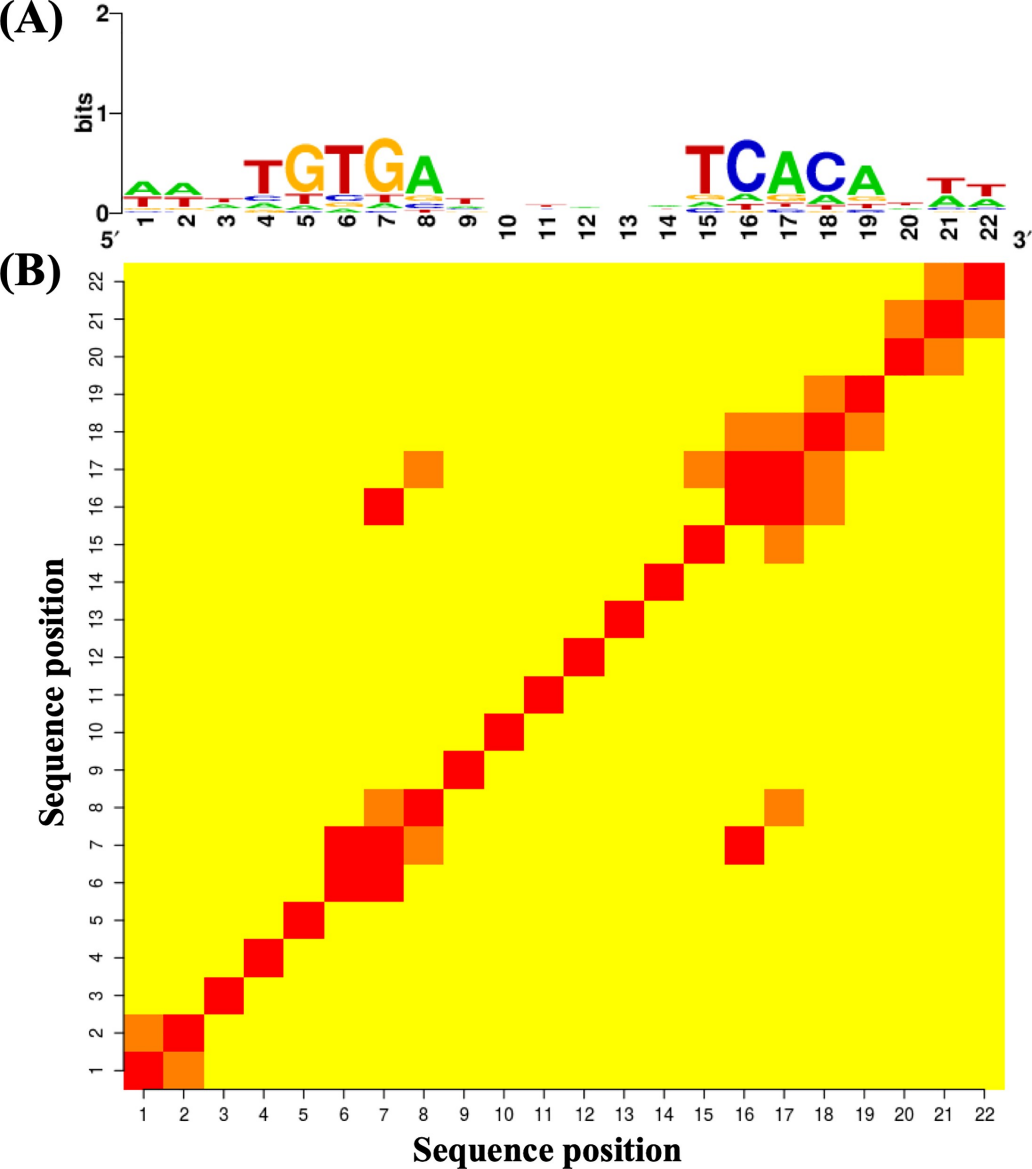
Sequence logo and heat map of CRP. Out of 374 CRP motifs, 308 sequences with the 22-bp motif were used. (A) Sequence logo for CRP using 308 sequences [34]. (B) Heat map of CRP.

CRP is one of the seven main transcription factors that influences transcriptional networks in *E*. *coli* [33]. It has been shown that there are interdependencies among neighboring DNA bases in CRP binding sites [29]. More than 300 binding sites for CRP have been registered in Regulon DB as “TF binding sites” (Release: 9.4) [32]. The CRP binding sites are separated by a 6-nt gap (Fig 1A). We measured the interdependency of CRP using the mutual information proposed by Salama and Stekel [29]. Strong correlations between neighboring bases were observed, for example, among positions 1, 2, and 6–8, and among positions 16–19 (Fig 1B). In addition, we observed the higher mutual information between the distant positions in 7, 16 and 8, 17 among the palindromic positions, followed by the position in 6 and 19. This suggests that the palindromic features of CRP binding sites would be incomplete.

### Performance for CRP dataset

**Fig 2:**
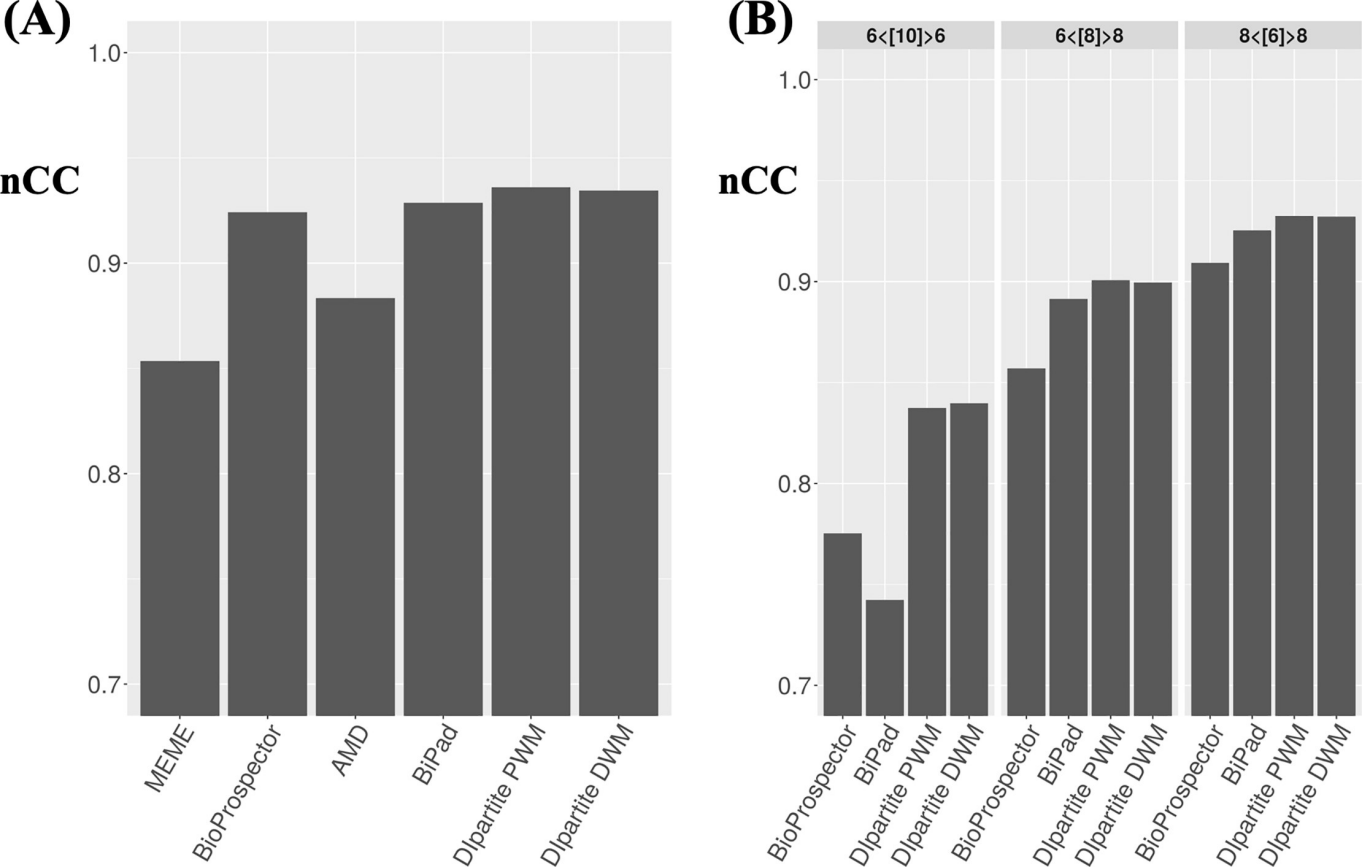
The performance comparison for 323 CRP sequences. The combined *nCC* values were plotted. (A) Summary of the results for searching the one-block motif, i.e., the 22 bp motif, by MEME, BioProspector, AMD, BiPad, DIpartite PWM and DIpartite DWM. (B) Summary of the results for searching the bipartite motifs, i.e., 6<[10]>6, 6<[8]>8, and 8<[6]>8, by BioProspector, BiPad, DIpartite PWM and DIpartite DWM.

We evaluated the performance of DIpartite by using the TF binding sites of CRP. Out of 374 sequences of CRP binding sites, 323 unique sequences were used as the test dataset. Jensen and Liu (2004) analyzed the CRP binding sites as a bipartite motif and proposed the consensus sequence, tGTcA<6,8>CAcattt [19,35]. We conducted motif prediction by using MEME (ver. 5.0.2), BioProspector (release 2), AMD, BiPad (ver. 2), and DIpartite for these 323 sequences of CRP binding sites (Fig 2A). DIpartite with the “PWM” or “DWM” options is referred to as DIpartite PWM or DIpartite DWM, respectively. Although DIpartite PWM performed best among the tested software for the one-block model, namely, the 22-bp motif, the performance was comparable among MEME, BioProspector, BiPad, and DIpartite. AMD exhibited a combined *nCC* value of less than 0.9. We assessed the performance of DIpartite by randomly sampling 100 datasets with 100 sequences from the CRP binding sites. DIpartite DWM slightly outperformed other tested tools for 100 datasets (S2A Fig). In addition, we tested the running time by using the CRP dataset. Although BioProspector was the fastest software among tested software, DIpartite was comparable with BiPad (S3 Fig).

For the bipartite motif, we compared BioProspector, BiPad, DIpartite PWM, and DIpartite DWM (Fig 2B). The performance of searching the bipartite motifs was lower than that of searching the one-block model, i.e., 0.936 by DIpartite PWM. For all three types of the bipartite motifs, DIpartite PWM and DIpartite DWM were superior to BioProspector and BiPad. DIpartite DWM was superior to DIpartite PWM in the case of 6<[10]>6. We conducted the performance comparison by using 100 datasets with 100 sequences (S2B Fig). DIpartite PWM outperformed other tested tools. Although the implementation of DIpartite PWM is similar to that of BiPad, DIpartite PWM slightly outperformed BiPad. This might be because DIpartite takes into consideration the background sites (not motif sites) unlike BiPad, that is, *b*(*x*) in Eq (2). Taking the findings together, DIpartite successfully detected the binding sites of the one-block or bipartite motifs.

### Performance for human dataset

**Fig 3:**
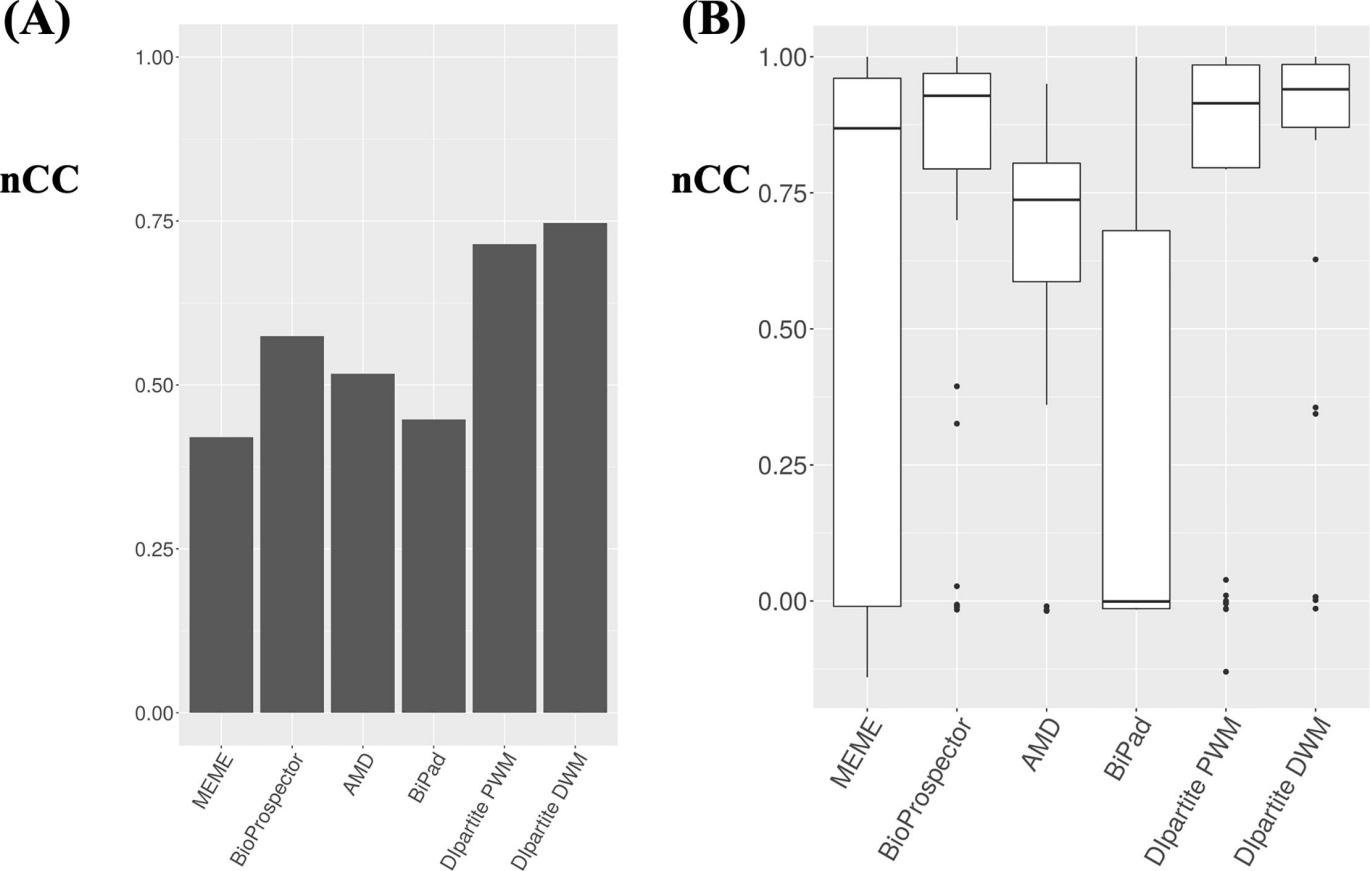
The performance comparison for human promoter datasets. (A) Summary of the results of all 40 human promoter datasets. The combined *nCC* values were calculated by using a total of 3,054 sequences. (B) Boxplots of the *nCC* values for each 40 human promoter datasets. All values are shown in S1 Table.

We selected the human promoter sequences as bipartite motifs with constant gaps in eukaryotes [15]. Of 1,460 motifs, 46 motifs with gaps larger than 4 nt were filtered. The promoter sequences around the positions of each motif (500 bp upstream to 500 bp downstream) were retrieved as the target sets. Since AMD did not detect any motifs for six motifs, namely, RGGANNNNNAKTCC (54 sequences), RKCTGNNNNNRMTTA (21 sequences), TTGRNNNNNNTCCAR (21 sequences), YMATCNNNNNGCGM (50 sequences), YTGGANNNNNNYCAA (26 sequences), and YTTGRNNNNNNGCCNR (50 sequences), these were excluded, and 40 datasets were evaluated for the performance of DIpartite. We assessed the performance for 40 motif datasets (Fig 3A). DIpartite DWM exhibited the highest performance (50%), followed by DIpartite PWM (48%), BioProspector (38%), MEME (20%), BiPad (8%), and AMD (3%) (S1 Table), indicating that DIpartite performs equivalently to or better than the other tools for detecting dipartite motifs. In addition to the result of CRP 6<[10]>6, DIpartite DWM outperformed other tested tools, suggesting that DWM might improve the bipartite motif detection. Apparently, MEME and BiPad exhibited larger interquartile ranges (Fig 3B), indicating that these tools outperformed DIpartite for particular motifs, but were outperformed by it for the other motifs.

### Performance for sigma factor dataset

**Fig 4:**
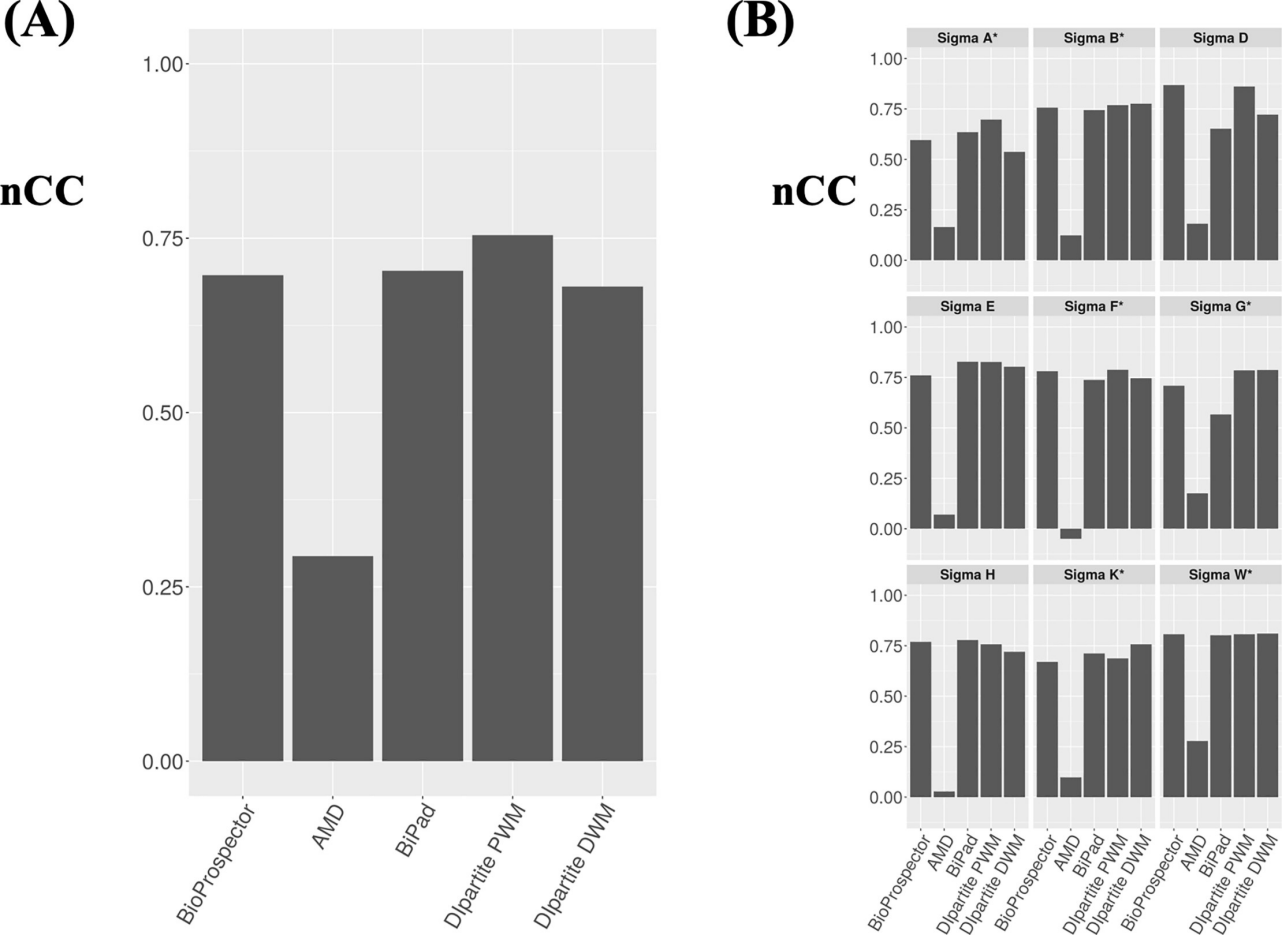
The performance comparison for *B*. *subtilis* datasets. (A) Summary of the results of all sigma datasets. (B) Summary of the results of each sigma datasets. σ^A^, σ^B^, σ^D^, σ^E^, σ^F^, σ^G^, σ^H^, σ^K^, and σ^W^ consist of 344, 64, 30, 70, 25, 55, 25, 53, and 34 sequences, respectively. The asterisks indicate if DIpartite performed better than BioProspector, AMD, and BiPad.

We compared the performance of DIpartite with those of BioProspector, AMD, and BiPad for bipartite motifs with variable gaps. We adopted the nine bipartite sigma transcription factors in *B*. *subtilis*, namely, σ^A^ (344 sequences), σ^B^ (64 sequences), σ^D^ (30 sequences), σ^E^ (70 sequences), σ^F^ (25 sequences), σ^G^ (55 sequences), σ^H^ (25 sequences), σ^K^ (53 sequences), and σ^W^ (34 sequences) from DBTBS [7] as the test datasets (Fig 4A). DIpartite PWM performed better than BioProspector, BiPad, AMD, and DIpartite DWM for six sigma factors, with the exceptions being σ^D^, σ^E^ and σ^H^ (Fig 4B). While the performance of DIpartite PWM was excellent for two sigma factors (σ^A^ and σ^F^), that of DIpartite DWM was remarkable for four sigma factors (σ^B^, σ^G^, σ^K^, and σ^W^). AMD exhibited relatively low *nCC* values for all nine datasets (Fig 4A), unlike the results for human promoter sequences, suggesting that the variable gap lengths could affect its performance. This is reasonable because AMD was developed for detecting bipartite motifs with constant gaps. AMD with the option “-T 1” did not detect any motifs for two sigma datasets, i.e., σ^E^ and σ^F^.

**Fig 5:**
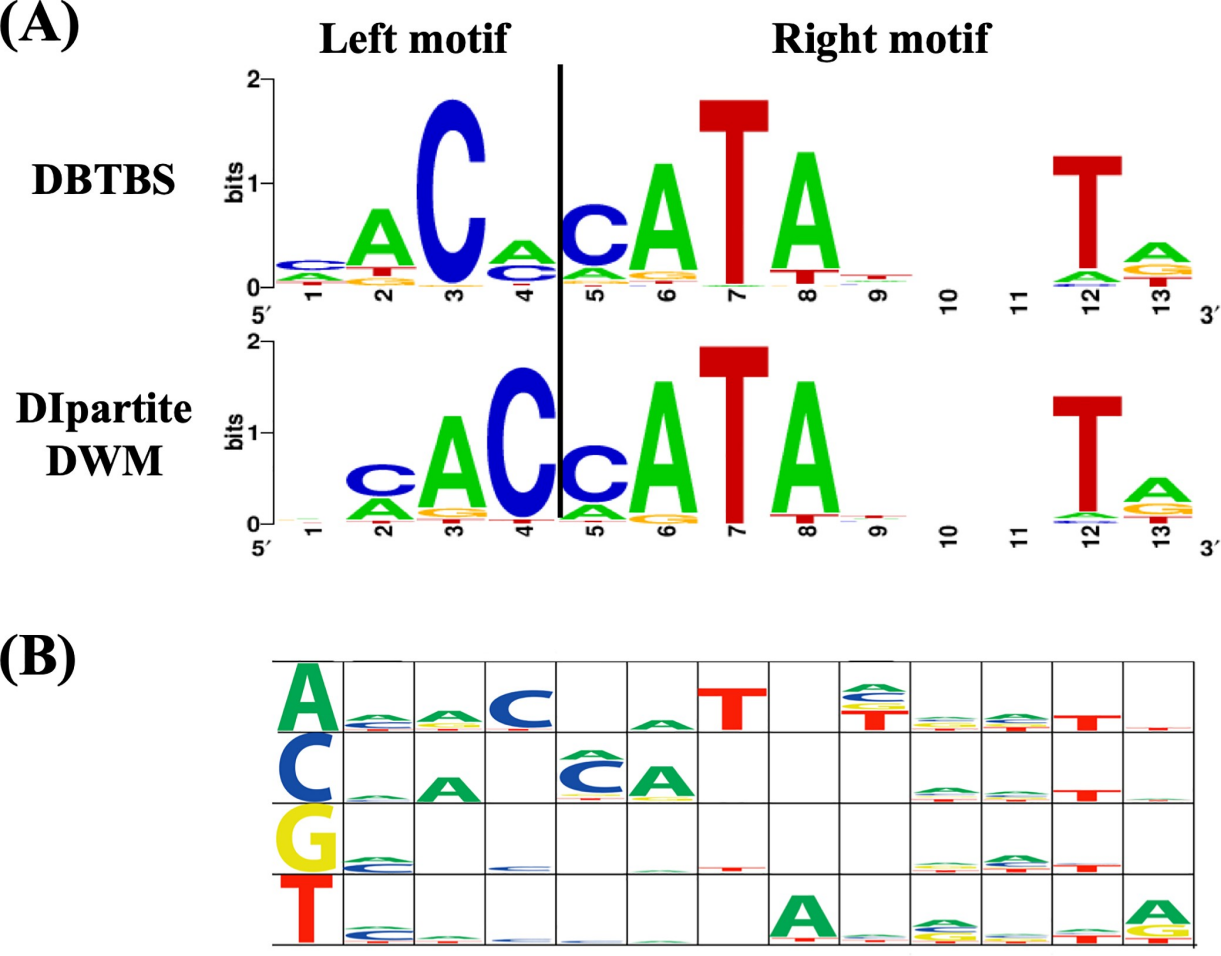
Sequence logo for σ^K^ by DIpartite DWM. (A) Sequence logos generated by DBTBS and DIpartite DWM. The border between the left and right motifs, i.e., position 4, 5, is indicated as the vertical line. (B) Sequence logo for the probability of each dinucleotides. One base before was depicted in first column. Size of each logo was proportional to the probability of dinucleotides.

Among four sigma factors with the highest performance coefficients for DIpartite DWM, the *nCC* value for σ^K^ was greatly improved by DIpartite DWM, namely, to 0.757, indicating the presence of base interdependencies in the motif of σ^K^. We observed that the left motif of DIpartite DWM was shifted and “AC” was more over-represented, indicating that the left motif of σ^K^ might be improved. Position 7 was “T” in all 53 sequences (Fig 5A), consistent with the known motif in DBTBS. Similarly, the highest frequencies of the dinucleotides “AT” and “TA” were observed at positions 6 and 7, and 7 and 8, respectively (Fig 5B).

The *nCC* value of σ^A^ was greatly improved by DIpartite PWM, namely, to 0.697. While the sequence logo generated from the result of BioProspector was similar to that generated from the result of DIpartite DWM, those of BiPad and DIpartite PWM was different from them (S4 Fig). In particular, DIpartite PWM exhibited the conserved base “T”, at position 1. This result is consistent with the motif TTGACA<>tgnTATAAT proposed by DBTBS [7]. DIpartite PWM showed the sequences with minimum entropy.

**Fig 6:**
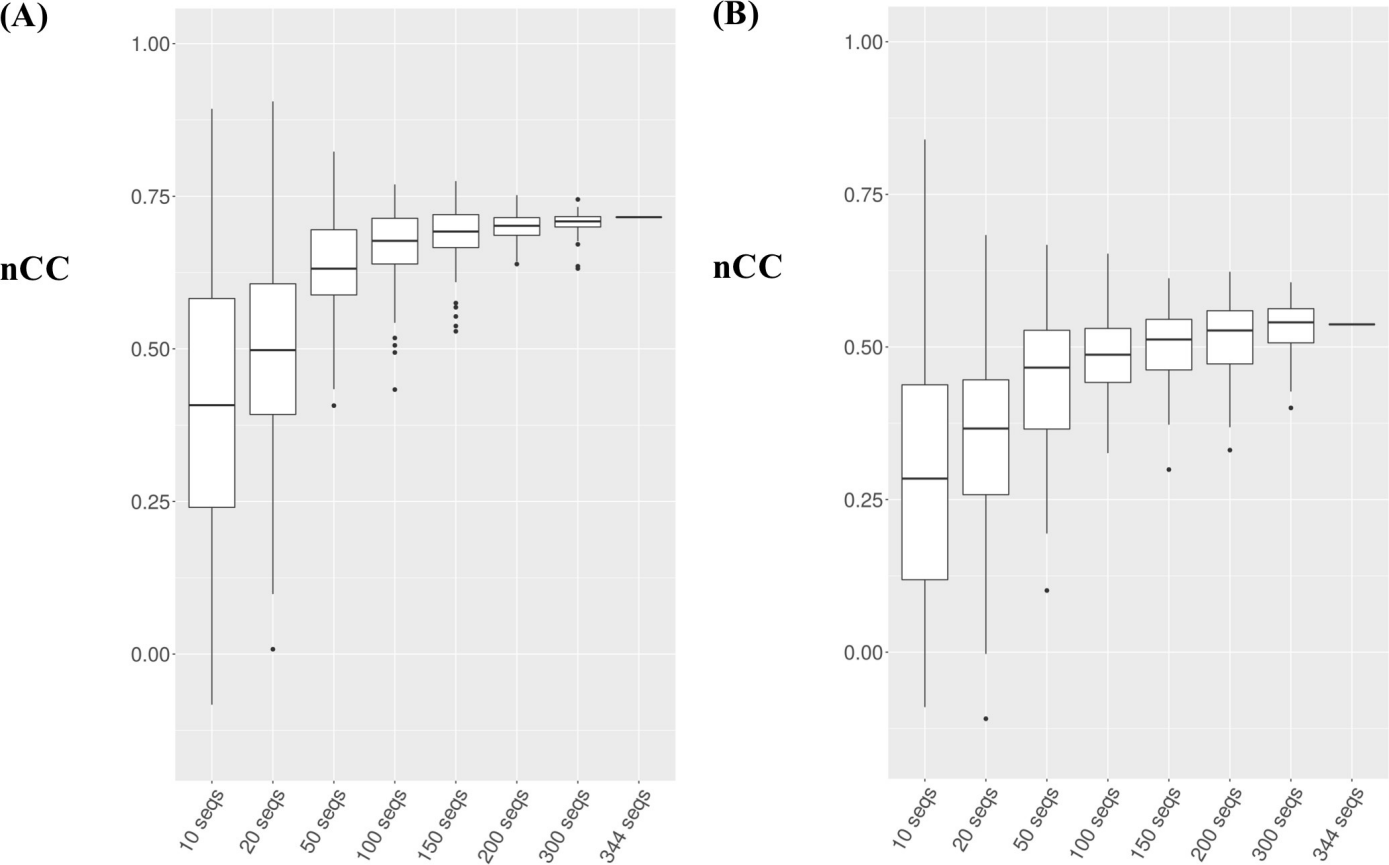
**The performance of DIpartite: (A) PWM; (B) DWM.** 100 datasets were generated by sampling of the σ^A^ dataset. The sizes of the dataset were 10, 20, 50, 100, 150, 200, 300 sequences.

We assessed the performance of DIpartite DWM in terms of the sizes of the input datasets. By randomly sampling the sequences of σ^A^ in *B*. *subtilis*, we generated 100 datasets for each including 10, 20, 50, 100, 150, 200, and 300 sequences (Fig 6). Upon increasing the size of the datasets, DIpartite PWM and DWM exhibited better performance. Notably, DIpartite underperformed for the datasets with 10 and 20 sequences, suggesting that DIpartite could perform well for data including more than 50 sequences. The variances of DIpartite PWM for the datasets with 200 and 300 sequences were relatively smaller than those of DIpartite DWM. One potential reason for this is that DWM consists of the frequencies of 16 dinucleotides (Eq 3).

### Performance for the dataset with noise sequences

We assessed the performance for the datasets with noise sequences. DIpartite allows the users to search the motifs for the datasets with noise sequences, known as ZOOPS. We evaluated the accuracy of detecting noise sequences by using the datasets with noise sequences. We chose the CRP datasets and human dataset as the test datasets of the one- and two-block motifs. We compared the performance of noise detection by DIpartite with that by MEME for the CRP datasets (Table 1). DIpartite exhibited the TPRs (true positive rate), i.e., 0.835, 0.863, and 0.876 for the datasets with 25%, 50%, and 100% noise sequences, respectively. This indicates that DIpartite ZOOPS could be well tolerated with the noise sequences. Indeed, MEME exhibited the lower FPRs, but lower TPRs, suggesting that DIpartite ZOOPS would be comparable with MEME ZOOPS.

**Table 1:** The performance of noise detection for the one-block motif.

	MEME			DIpartite		
	CRP_25	CRP_50	CRP_100	CRP_25	CRP_50	CRP_100
**FPR**	0.061	0.030	0.024	0.172	0.172	0.167
**TPR**	0.798	0.777	0.739	0.835	0.863	0.876

Noise sequences were sampled from the genome sequence of *E*. *coli*. CRP_25 consists of 323 CRP and 81 (25%) noise sequences. CRP_50 consists of 323 CRP and 162 (50%) noise sequences. CRP_100 consists of 323 CRP and 323 (100%) noise sequences.

TPR: True positive rate, FPR: False positive rate.

Finally, we compared the performance of noise detection for the two-block dataset, i.e., RYAAAKNNNNNNTTGW consisting of 44 sequences (S1 Table). BioProspector (*nCC* = 1) and BiPad (*nCC* = 1) outperformed DIpartite PWM (*nCC* = 0.914). Increasing the noise sequences, BioProspector and BiPad exhibited lower *nCC* values (Table 2). DIpartite exhibited higher *nCC* values even adding the noise sequences, suggesting that DIpartite could work well for both one- and two-block motifs with noise sequences.

**Table 2:** The performance of noise detection for the two-block motif.

	TF_0	TF_25	TF_50	TF_100
**BioProspector**	1	0.907	−0.16	−0.16
**BiPad**	1	1	1	−0.16
**DIpartite PWM**	0.914	1	1	1

The combined *nCC* values were indicated. Noise sequences were sampled from the genome sequence of human. TF_0 consists of 44 RYAAAKNNNNNNTTGW sequences. TF_25 consists of 44 RYAAAKNNNNNNTTGW and 11 noise sequences. TF_50 consists of 44 RYAAAKNNNNNNTTGW and 22 noise sequences. TF_100 consists of 44 RYAAAKNNNNNNTTGW and 44 noise sequences.

## Conclusions

We have developed DIpartite for the detection of TFBSs, consisting of bipartite motifs. DIpartite enables *ab initio* prediction of conserved motifs based on not only PWM, but also DWM. We evaluated the performance of DIpartite compared with freely available tools, namely, MEME, BioProspector, AMD, and BiPad. Both DIpartite PWM and DWM performed equivalently to or better than these alternatives, especially in the case of the bipartite motifs with variable gaps, like for sigma factors in *B*. *subtilis*. The prediction of σ^K^ was greatly improved by taking into consideration base interdependencies. DIpartite is available for use at https://github.com/Mohammad-Vahed/DIpartite.

## Supporting information

S1 FigFlowchart of DIpartite.The input data is the sequence file including *N* sequences. DIpartite proposes bipartite motifs based on PWM or DWM. Each iteration starts from randomly generated positions. The convergence of each iteration is judged by the differences of the entropy, that is, ε. We set *ε* = 10^−8^. E_*i*_ and E_*i*−1_ correspond to the *i*th and *i*−1th entropy, i.e., ${IC}_{M_{LR}}$ (Eq 2), respectively.(TIFF)

S2 Fig The performance comparison for 100 CRP datasets. 100 datasets consisting of 100 sequences were generated by randomly sampling the CRP datasets. (A) Summary of the results for searching the one-block motif, i.e., the 22 bp. (B) Summary of the results for searching the bipartite motifs, i.e., 6<[10]>6, 6<[8]>8, and 8<[6]>8.
(TIFF)

S3 Fig Running times. The datasets consisting of 20, 50, 100, 200, 500 and 1,000 sequences were generated by randomly sampling the CRP sequences. X-axis and Y-axis correspond to the number of sequences, and the running time [s] on a log scale. BioProspector (designated as Bio), BiPad, DIpartite PWM (designated as PWM), and DIpartite DWM (designated as DWM) were tested.
(TIFF)

S4 Fig Sequence logos for σ^A^ from the results of (A) BioProspector, (B) BiPad, (C) DIpatrite PWM, and (D) DIpartite DWM.
(TIFF)

S1 Table The performance comparison for 40 motifs in human. (XLSX)
