# Supplementary figures and images for: DIpartite: A tool for detecting bipartite motifs by considering base interdependencies
Source: PLoS One. 2019 Aug 30;14(8):e0220207. doi: 10.1371/journal.pone.0220207 (PMC6716629; doi:10.1371/journal.pone.0220207)

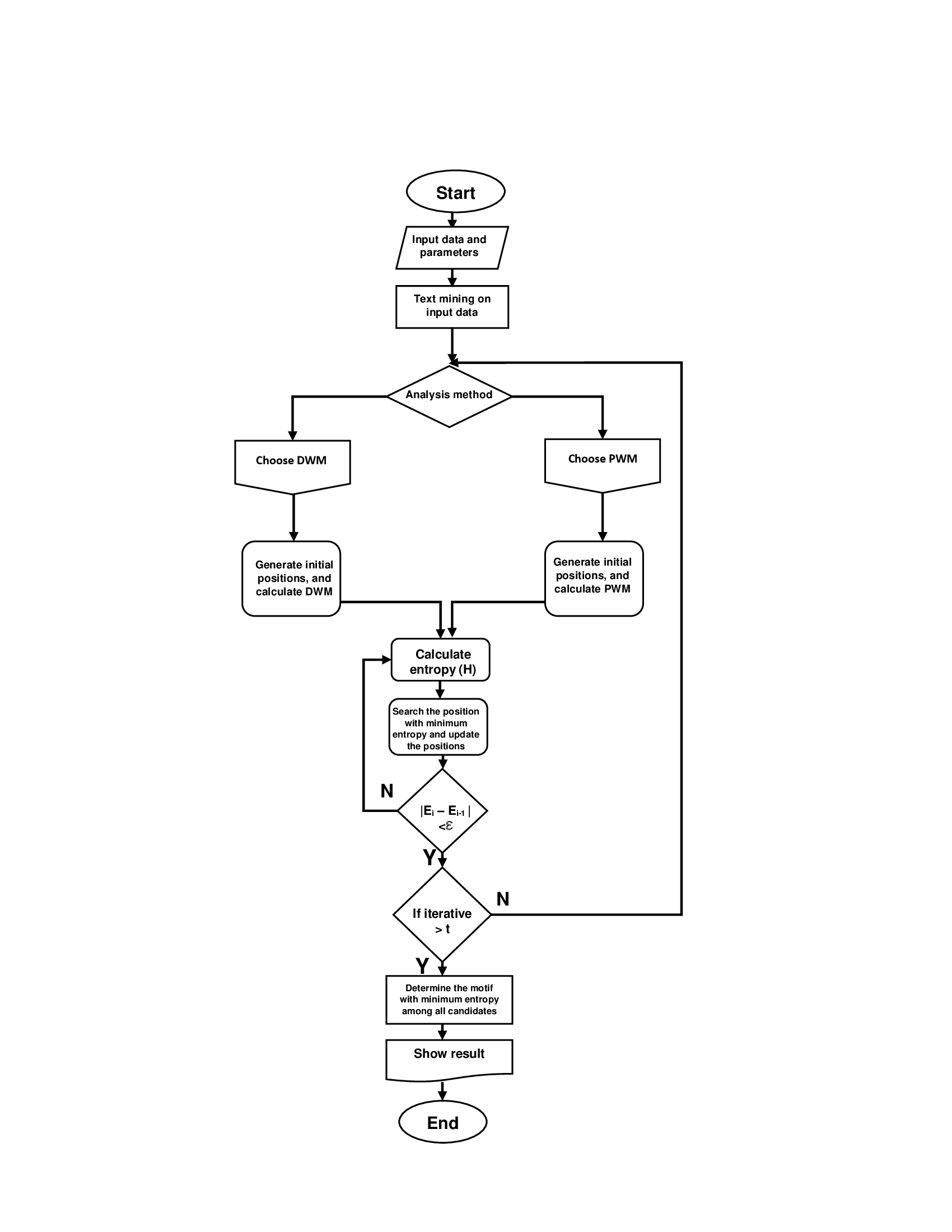

Supplement: S1 Fig — The input data is the sequence file including N sequences. DIpartite proposes bipartite motifs based on PWM or DWM. Each iteration starts from randomly generated positions. The convergence of each iteration is judged by the differences of the entropy, that is, ε. We set ε = 10−8. Ei and Ei−1 correspond to the ith and i−1th entropy, i.e., ICMLR (Eq 2), respectively. (TIFF) [file pone.0220207.s001.tiff]

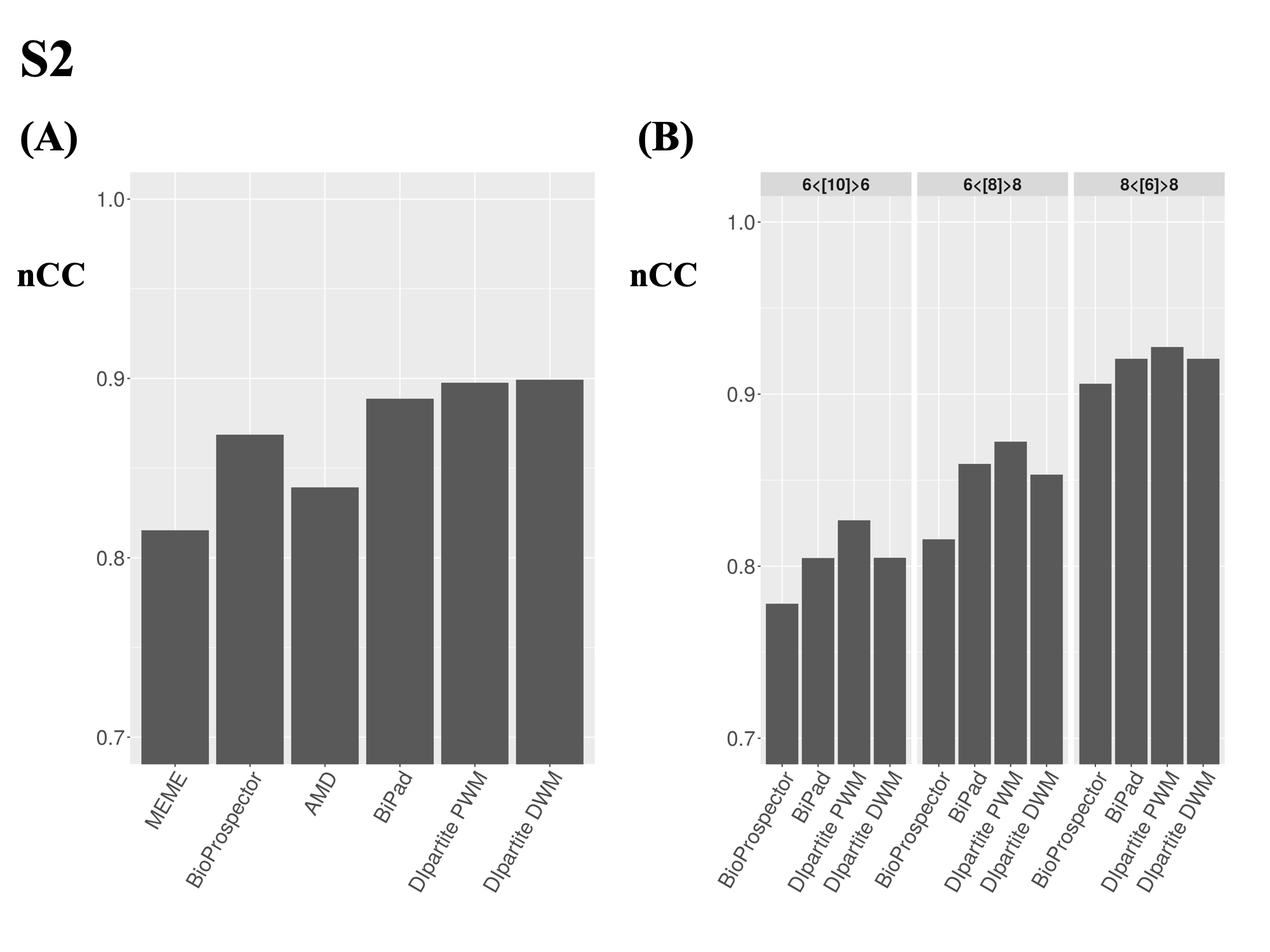

Supplement: S2 Fig — 100 datasets consisting of 100 sequences were generated by randomly sampling the CRP datasets. (A) Summary of the results for searching the one-block motif, i.e., the 22 bp. (B) Summary of the results for searching the bipartite motifs, i.e., 6<[10]>6, 6<[8]>8, and 8<[6]>8. (TIFF) [file pone.0220207.s002.tiff]

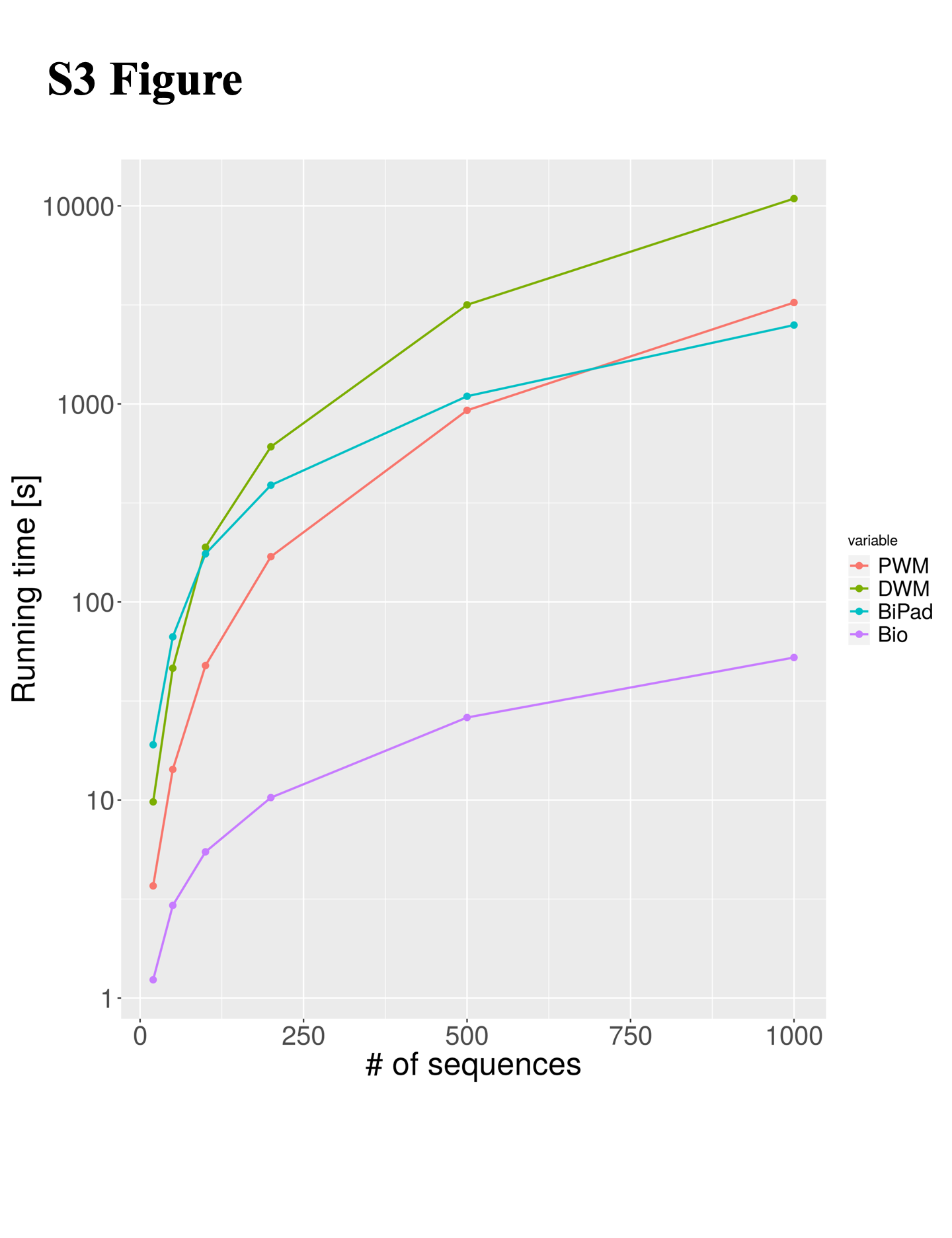

Supplement: S3 Fig — The datasets consisting of 20, 50, 100, 200, 500 and 1,000 sequences were generated by randomly sampling the CRP sequences. X-axis and Y-axis correspond to the number of sequences, and the running time [s] on a log scale. BioProspector (designated as Bio), BiPad, DIpartite PWM (designated as PWM), and DIpartite DWM (designated as DWM) were tested. (TIFF) [file pone.0220207.s003.tiff]

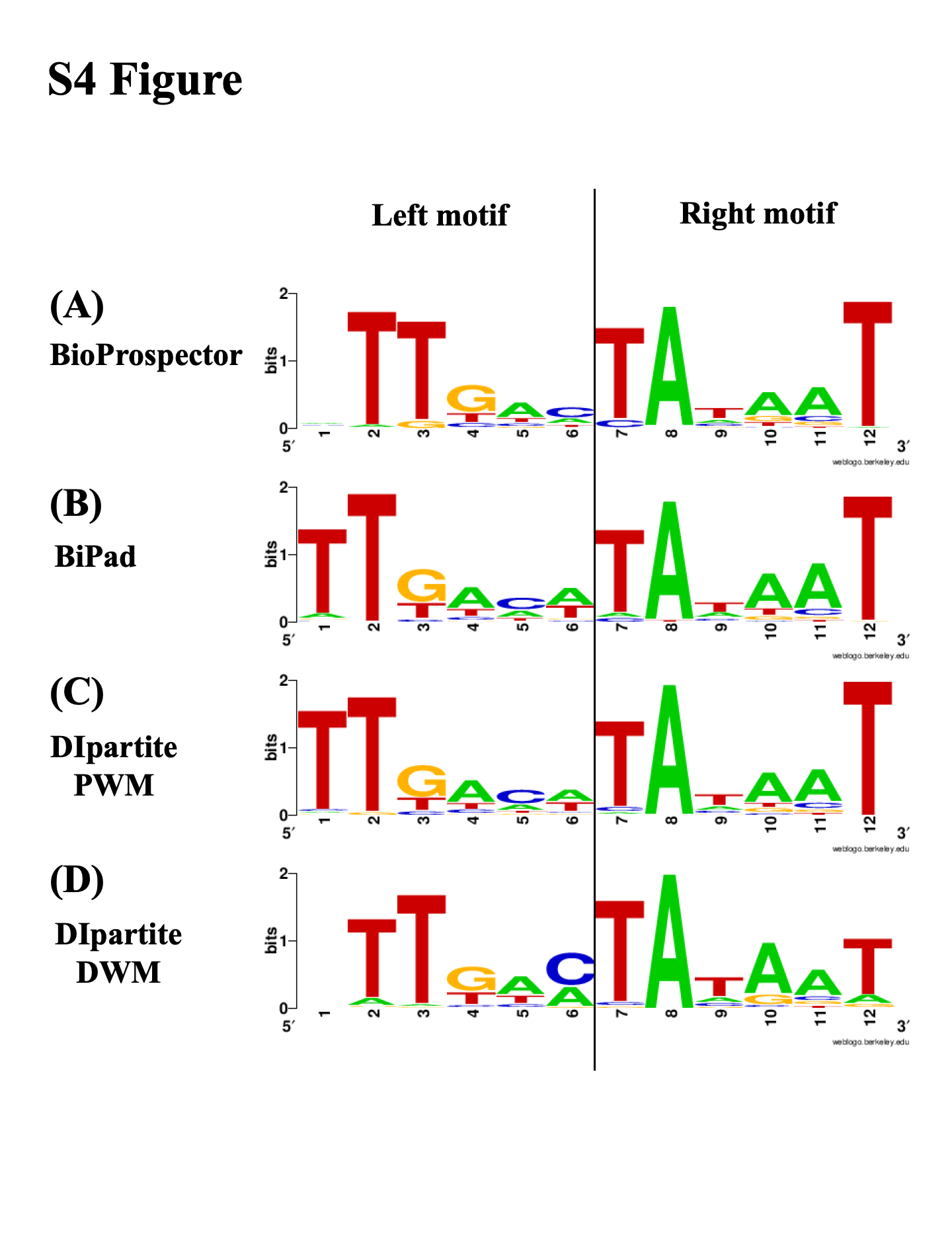

Supplement: S4 Fig — Sequence logos for σA from the results of (A) BioProspector, (B) BiPad, (C) DIpatrite PWM, and (D) DIpartite DWM. (TIFF) [file pone.0220207.s004.tiff]
